# 

**Published:** 1884-07

**Authors:** 


JULY, 1884.

NUMBER 12, VOLUME XXIII.

### THE BUFFALO Medical and Surgical Journal.

LIBRARY INDEXED S.G.O.

EDITORS:

PUBLISHED BY THE MEDICAL JOURNAL ASSOCIATION.

No. 5 WEST CHIPPEWA ST.

Entered at the Post-Office at Buffalo, N. Y., as Second-class Mail Matter.

